# Complement response to burn injury: systematic review and meta-analysis of patient and animal studies

**DOI:** 10.3389/fimmu.2026.1793945

**Published:** 2026-02-25

**Authors:** Patrick P. G. Mulder, Marit van Hooren, Roland V. Bumbuc, Carlijn R. Hooijmans, H. Ibrahim Korkmaz, Bouke K. H. L. Boekema

**Affiliations:** 1 Burn Research Lab, Alliance of Dutch Burn Care (ADBC), Beverwijk, Netherlands; 2 Department of Plastic, Reconstructive and Hand Surgery, Amsterdam UMC, Amsterdam, Netherlands; 3 Amsterdam Movement Sciences, Tissue Function and Regeneration, Amsterdam, Netherlands; 4 Computational Science Lab, Informatics Institute, University of Amsterdam, Amsterdam, Netherlands; 5 Department of Molecular Cell Biology and Immunology, Amsterdam Infection and Immunity (AII) Institute, Amsterdam UMC, Amsterdam, Netherlands; 6 Meta-Research Team, Department of Anesthesiology, Pain and Palliative Medicine, Radboud University Medical Center, Nijmegen, Netherlands; 7 Alliance of Dutch Burn Care, Burn Center and Department of Plastic and Reconstructive Surgery, Red Cross Hospital, Beverwijk, Netherlands

**Keywords:** blood, burn injury, complement, immune response, inflammation, skin

## Abstract

**Background:**

Burns often induce a profound inflammatory response that contributes to immune dysfunction, tissue damage, and adverse clinical outcomes. Activation of the complement system plays a crucial role in this response, yet findings across different studies are heterogeneous and lack quantitative synthesis. Therefore, we performed a systematic review and meta-analysis to characterize the overall and temporal dynamics of complement activation following burn injury.

**Methods:**

PubMed and Embase were searched on February 20th, 2025, for human and animal studies reporting quantitative data on complement factors after cutaneous burn injury. Meta-analyses were conducted for the reported outcomes. Subgroup analyses were performed for predefined time intervals (post burn days 0-1, 2-4, 5-9, 10-14, 15-21, versus >21). Risk of bias was assessed using SYRCLE and ROBANS-II tools.

**Results:**

A total of 110 studies were included in the review, of which 73 were eligible for meta-analysis. The included studies encompassed diverse animal models and human patient cohorts with wide variation in burn size, depth, aetiology, and sampling time points. Across both animal and human studies, substantial underreporting of key methodological details resulted in predominantly unclear or high risk of bias, limiting interpretability and reproducibility. Overall analyses revealed significant increases in C3a, C3b, C5a, factor B, and membrane attack complex (MAC), while alternative pathway activity, C3 conversion, C4, and properdin were reduced. Total complement activity and C3 were not significantly altered in overall analysis. Longitudinal analyses demonstrated a dynamic response: total complement, C3, and C4 were markedly reduced during the first 24 hours, followed by normalization and subsequent elevation of C3 from 10 days after injury.

**Conclusions:**

Burn injury is associated with a time-dependent alteration of complement activity characterized by early consumption followed by sustained activation that likely contributes to prolonged inflammation and impaired healing. Our findings provide guidance for complement involvement in burn pathology and support further investigation of time-specific complement-targeted therapeutic strategies.

**Systematic review registration:**

https://www.crd.york.ac.uk/PROSPERO/, identifier CRD420250510109.

## 1 Introduction

Burn injury triggers a profound inflammatory response, and patients often develop immune disturbances that contribute to extensive tissue damage, metabolic stress, and poor outcomes (1). A key driver of this response is the activation of the complement system (2). Complement factors, together with cytokines and immune cells, initially function to contain tissue injury and promote repair but can rapidly derail into a dysregulated response associated with shock, coagulopathy, immunosuppression, and multi-organ failure (3).

**Figure 1:**
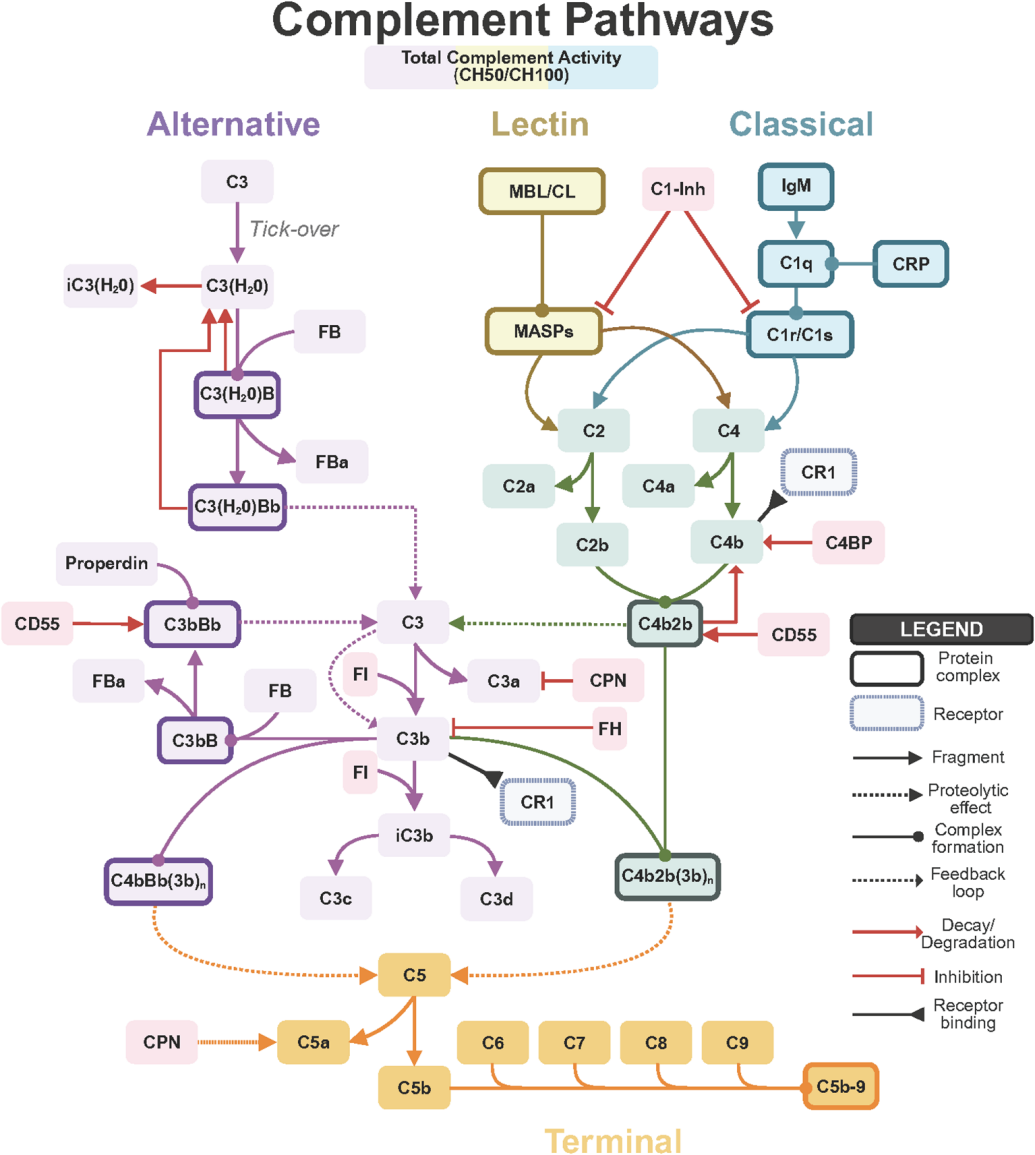
The complement activation pathways - classical (blue), lectin (tan/yellow), and alternative (purple) – that converge on C3 cleavage. The classical pathway is activated by IgM and CRP binding to C1q, followed by C1r/C1s-mediated cleavage of C4 and C2, forming the C4b2b convertase. C1 inhibitor (C1-Inh) regulates C1 activation. The lectin pathway is triggered by MBL/CL binding, activating MASPs to cleave C4 and C2. The alternative pathway initiates through spontaneous C3 tick-over, forming C3(H_2_O) that binds Factor B to generate C3(H_2_O)Bb. This cleaves C3 to produce C3b, which binds FB to form the amplification convertase C3bBb, stabilized by properdin and negatively regulated by CD55. C3 cleavage generates anaphylatoxin C3a and opsonin C3b. Factor I (FI), with cofactors Factor H (FH) and C4BP, degrades C3b and C4b into iC3b, C3c, and C3d fragments. Carboxypeptidase N (CPN) inactivates C3a. C5 convertases from all pathways cleave C5 into anaphylatoxin C5a and C5b, initiating terminal pathway (orange) assembly of C6-C9 into the membrane attack complex (C5b-9/MAC). CR1 binds C3b/C4b for immune complex clearance. CH50/CH100 (top, multi-colored) measures total complement activity.

The complement system comprises three activation pathways, classical, lectin, and alternative, that converge on C3 cleavage and subsequent formation of inflammatory mediators and lytic complexes (**Figure 1**) (2, 4, 5). The classical pathway is activated when natural IgM antibodies and C-reactive protein (CRP) recognize heat-induced neoepitopes on damaged tissue, whereas the lectin pathway is triggered by binding of mannose-binding lectin (MBL) to exposed carbohydrate structures (6, 7). Critically, the alternative pathway provides an amplification loop after burn injury and accounts for the majority of C3 activation (8). C3 is the central complement component where all three pathways converge; its cleavage generates anaphylatoxin C3a and opsonin C3b, which deposits on damaged tissue to amplify the cascade and facilitate phagocytosis (8, 9). Subsequent C5 activation generates anaphylatoxin C5a that drives inflammation, neutrophil recruitment, and vascular permeability, and ultimately leads to formation of the membrane attack complex (MAC; C5b-9), which lyses target cells (2). Complement components involved in pathway initiation and amplification, such as C4 (forming the classical/lectin C3 convertase C4b2a), factor B (forming the alternative-pathway convertase C3bBb), and properdin (which stabilizes this convertase), further shape the magnitude and duration of complement activity.

Previously an extensive overview displaying the dynamics of immune cells and soluble immune mediators, including cytokines, chemokines, and growth factors, following burn injury (3, 4) was created (10, 11). To develop a more complete characterization of the post-burn inflammatory response, the present systematic review and meta-analysis extend this dataset with complement factors. By integrating findings from both animal and human studies, we aim to elucidate changes in complement activity after burns. Numerous studies have examined how burn injury affects individual complement components, each playing distinct mechanistic roles in the inflammatory cascade. However, these findings are scattered across decades of research using heterogeneous models, time points, sampling strategies, and analytical methods. As a result, a coherent and quantitative understanding of complement alterations after burn injury is still lacking. This gap limits our ability to understand the trajectory of immune dysregulation, its relationship to clinical outcomes, and the potential for complement-targeted therapies in critical care.

A unified dataset incorporating complement components alongside broader immune parameters will improve our understanding of burn-induced immune dysregulation and may guide the development of prediction models and therapeutic strategies to prevent complications such as sepsis and impaired wound healing (12). Accordingly, the aim of this systematic review is to comprehensively evaluate how complement activation is altered after burn injury and to determine its contribution to inflammatory responses and clinical outcomes.

## 2 Methods

### 2.1 Study protocol and eligibility criteria

For this review we investigated how complement factors (outcome) were affected by burn injury (intervention) in burn patients and animal models (population) compared to control groups (comparison). The associated review protocol was established beforehand and registered at the International Prospective Register of Systematic Reviews (PROSPERO) (13) under number CRD420250510109 (https://www.crd.york.ac.uk/PROSPERO/view/CRD420250510109).

### 2.2 Search strategy

The search was performed using PubMed and Embase, with a final update on February 20^th^, 2025. The search strategy included keywords to include records about ‘burn injuries’ combined with records about ‘complement system’ (**Supplementary File 1**). No restrictions were applied regarding language or publication date. Search results were combined, and duplicate records were removed using EndNote software (X9, Clarivate Analytics, London, United Kingdom).

### 2.3 Study selection

Studies were selected by PPGM, MvH and BKHLB in a blinded fashion using Rayyan software (Rayyan Systems, Cambridge, MA) (14), over two rounds. In round 1, the title and abstract screening, we selected studies that involved mammals including humans with skin burns and that contained primary data about any complement factor. We excluded reviews, posters, and conference abstracts. Discrepancies between reviewers were discussed and re-evaluated in the second round. Articles that were neither in English nor Dutch were translated into English using Google Translate. Full text versions were obtained through VU university licenses or by ordering through VU university library Amsterdam. Studies for which the full-text could not be obtained through these methods were excluded from the review.

During round 2, the full-text screening, studies that used an appropriate control group (i.e. sham controls, uninjured group, baseline values, or samples of uninjured tissue from same group) or studies which reported reference values were selected. We excluded studies that involved frost/low-temperature, chemical, radiation or electrical injuries or studies with co-interventions (for all subjects) that are known to interfere strongly with the function of the immune system, such as infection or administration of pro- or anti-inflammatory therapeutics. Information about therapeutic interventions and complement factors investigated in those studies were reported. Discrepancies in this round were solved by careful evaluation and discussion or, when necessary, by consultation of a third reviewer.

### 2.4 Study characteristics

Independently, PPGM, MvH, RVB, HIK and BKHLB extracted the study characteristics, including animal/subject details (species, strain, age, sex and weight), burn method/case details (burn agent, burn size (<5, 5–20 or >20), burn temperature, burn time, burn depth and injury site) and experimental set up (type of comparison, resuscitation and detection method) from a subset of studies. All extracted data was checked by at least one additional reviewer.

### 2.5 Study quality and risk of bias assessment

The reporting of any form of randomization or blinding and the presence of a conflict-of-interest statement was scored for all the included studies by either PPGM, MvH, RVB, HIK or BKHLB and at least 10% of those were checked by a second reviewer. Full risk of bias (RoB) assessment for animal studies was conducted using SYRCLE’s Risk of Bias tool (15), independently by MvH and BKHLB on all animal studies that included a separate control or sham group (n = 26). The reporting of the following baseline characteristics was evaluated: species, sex, age, or weight (a reported range<10% was considered low RoB). To check the completeness of outcome reporting, we evaluated the number of animals in the method and results section for each experiment and outcome. All baseline-controlled animal studies (n = 10) were scored separately, as only items 7, 8, and 9 of the SYRCLE’s RoB tool applied these studies. RoB in human studies was assessed by HIK and BKHLB using the ROBANS-II assessment tool on a random sample of 25 studies (16). In the case of discrepancies between the two reviewers that were not dismissible, PPGM was consulted as the decisive third reviewer. See **Table 1** for the studies used for the risk of bias assessment.

**Table 1:** Overview of the studies used for risk of bias assessment, systematic review and meta-analysis, and the studies that were not accessible.

Category	References
RoB assessment for animal studies with uninjured control group	26 studies (17–42)
RoB assessment for baseline-controlled animal studies	10 studies (43–52)
RoB assessment for human studies	Random sample of 25 studies (53–77)
Studies of which full texts could not be retrieved	54 studies (78–131)
Studies included in the systematic review	110 studies (9, 17–39, 41–60, 62–74, 76, 77, 132–182)
Studies included in the meta-analysis	73 studies (9, 18–22, 24, 26–28, 30–32, 34, 35, 37, 38, 41, 43–49, 51, 53, 54, 56, 57, 59, 60, 63, 65, 67, 69, 71, 72, 74, 132–134, 136–139, 142–145, 147–150, 153, 155–158, 161–165, 167–170, 176–180)

### 2.6 Outcome data extraction

All quantitative outcome measures related to the complement system, such as C3, C5 and CH50, in either blood or burn wound tissue (including blister fluid) that were assessed in at least 5 separate studies were collected (**Table 2**). The complete dataset is available upon request. PPGM, MvH, RVB, HIK and BKHLB independently extracted the outcome measures (mean outcome and SD/SEM, unit of measurement, number of animals/subjects) from a subset of studies. Extracted data was checked by at least one other reviewer. As a secondary outcome measure, the names of immunomodulatory treatments (including the administration route, studied species and analyzed outcomes) were collected for the included studies.

**Table 2:** Outcome measures and associated references used in systematic and/or meta-analysis.

Complement factor	References in systematic review	References used in meta-analysis
Alternative pathway (AP, AP50, AP100)	8 studies 18, 26, 27, 48, 63, 152, 153, 156	7 studies 18, 26, 27, 48, 63, 153, 156
C1	3 studies 25, 33, 148	<5 studies
C1inh (C1 inhibitor)	8 studies 66, 68, 140, 149, 152, 154, 166, 174	<5 studies
C1q	4 studies 57, 137, 152, 170	<5 studies
C1r	1 study 152	<5 studies
C1s	2 studies 149, 152	<5 studies
C2	4 studies 57, 137, 148, 152	<5 studies
C3	64 studies 17, 19, 20, 22, 23, 25, 26, 28, 31, 34, 39, 41, 43, 44, 46, 47, 49–52, 55–58, 62–65, 69–72, 76, 77, 136, 137, 140, 143, 144, 146–153, 155, 159–161, 165, 167–173, 175, 177, 178, 180, 182	37 studies 19, 20, 22, 26, 28, 31, 34, 41, 43, 44, 46, 47, 49, 51, 57, 63, 65, 69, 71, 72, 136, 137, 143, 144, 147–150, 153, 155, 161, 165, 167–170, 180
C3 conversion by cobra venom	3 studies 57, 136, 137	<5 studies
C3 conversion by inulin	5 studies 56, 57, 136–138	5 studies 56, 57, 136–138
C3a	10 studies 9, 32, 59, 67, 74, 145, 158, 162, 163, 179	10 studies 9, 32, 59, 67, 74, 145, 158, 162, 163, 179
C3a inactivator	2 studies 142, 147	<5 studies
C3b	6 studies 53, 54, 56, 133, 134, 142	6 studies 53, 54, 56, 133, 134, 142
C3b inactivator	3 studies 56, 57, 137	<5 studies
C3c	2 studies 157, 160	<5 studies
C3-C9	1 study 69	<5 studies
C3d complement factor 3d	4 studies 142, 176–178	<5 studies
C3d/C3 ratio	1 study 62	<5 studies
C4	29 studies 26, 43, 46, 47, 51, 57, 58, 63, 69, 71, 72, 132, 137, 140, 143, 146, 148, 149, 152, 153, 157, 159, 167, 169–171, 175, 180, 182	20 studies 26, 43, 46, 47, 51, 57, 63, 71, 72, 132, 137, 143, 148, 149, 153, 157, 167, 169, 170, 180
C4a	2 studies 59, 179	<5 studies
C4bp	1 study 152	<5 studies
C5	6 studies 25, 57, 137, 149, 152, 170	<5 studies
C5 conversion	1 study 137	<5 studies
C5a	11 studies 38, 59, 74, 139, 141, 145, 162–164, 171, 179	8 studies 38, 59, 74, 139, 145, 163, 164, 179
C6	2 studies 51, 152	<5 studies
C7	2 studies 44, 152	<5 studies
C8	4 studies 25, 26, 44, 152	<5 studies
C9	3 studies 25, 152, 181	<5 studies
CH50 inactivating capacity	1 study 45	<5 studies
Classical pathway	2 studies 156, 181	<5 studies
Complement CH50, CH100, total complement	33 studies 18, 20–26, 29, 35–37, 42, 45, 48, 51, 52, 55–58, 60, 63, 71–73, 137, 142, 152, 153, 171, 175, 181, 182	20 studies 18, 20–22, 24, 26, 35, 37, 45, 48, 51, 56, 57, 60, 63, 71, 72, 137, 142, 153
CPN Carboxypeptidase N	1 study 29	<5 studies
Factor B	11 studies 53, 54, 56, 57, 63, 69, 133, 134, 137, 152, 171	9 studies 53, 54, 56, 57, 63, 69, 133, 134, 137
Factor Ba	1 study 177	<5 studies
Factor H	1 study 152	<5 studies
Factor I	1 study 152	<5 studies
Lectin pathway	1 study 156	<5 studies
MAC membrane attack complex, terminal complement complex, TCC	6 studies 30, 48, 135, 145, 153, 156	5 studies 30, 48, 145, 153, 156
Properdin	7 studies 53, 54, 56, 57, 63, 69, 137	7 studies 53, 54, 56, 57, 63, 69, 137
Properdin convertase	1 study 69	<5 studies

A minimum of 5 articles was required for inclusion of a defined outcome measure in the meta-analysis. References that did not include a quantitative outcome measure or did not report essential study characteristics such as number of animals and standard deviation/error are shown in “References in systematic review” but were not used in meta-analysis.

Data from graphs were extracted using the digital ruler feature in ImageJ (version 1.53j, National Institutes of Health, Bethesda, MD) (183). Data presented as SEM were transformed to SD using the formula: SD = SEM * $\sqrt{number\;of\;animals}$. When studies used a relative expression (e.g., protein or mRNA expression level compared to uninjured group) and no SD/SEM was available, the SD/SEM of the burn-injured group was used as imputation for the uninjured group. In the absence of distributional information, we used the midpoint of reported reference ranges as the mean and half the range as SD, a conservative and transparent imputation strategy that avoids underestimating variability while enabling inclusion of studies with incomplete reporting. In case of missing data, such as the number of animals or SD, corresponding authors were contacted by email and ResearchGate, including a reminder after 2 weeks. After this, studies with missing data were excluded from the meta-analysis.

In the case of repeated measures of an experimental group within a time interval, the maximum effect size within that time interval was selected. When required, total body surface area was calculated using the reported area of the burn, weight (W) of the animals, and Meeh-Rubner’s formula ( $TBSA=\;\frac{area\;of\;burn}{K*W^{2/3}}$) (184). The following K values were used: 9.0 (mouse), 9.8 (rat), 10.0 (pig), 10.1 (dog), 10.4 (cat) 10.5 (guinea pig), and 12.0 (rabbit). When total body surface area was missing in included articles, it was estimated on the basis of the reported age and weight information available at Animal Resources Centre (https://www.ozgene.com), The Jackson Laboratory (https://www.jax.org/), and Roysfarm (https://www.roysfarm.com/). Animal age subgroups, young or adult, were based on the sexual maturity of the animals: adults were aged >12 weeks (hamster), >3 months (mouse), >6 months (rat, cat, pig or Guinea pig), >12 months (rabbit or dog), and >18 years for human. For wound depth, the following categories were used: superficial (first degree), partial-thickness (second degree), deep dermal (deep second degree), and full-thickness (third degree, severe burn injury).

### 2.7 Synthesis of results and meta-analysis

Meta-analyses were performed on overall outcome measures from which at least 5 separate studies were available. Data were analyzed using Comprehensive Meta-Analysis software (version 4; Biostat, Englewood, NJ), and the effect sizes were expressed as standardized mean difference (SMD; Hegdes’s g) of complement factor levels in burn-injured group compared to levels in uninjured group (baseline or uninjured comparison) with 95% confidence interval. A random-effects model was used in the analyses and the I^2^ statistic was used as a measure for statistical heterogeneity. Complement factors that were considered the same entity, such as MAC and TCC, were pooled (**Table 2**). Possible publication bias was explored using Duval and Tweedie’s trim-and-fill method (185).

### 2.8 Subgroup analyses

Predefined subgroup meta-analyses were performed on subgroups that consisted of at least 10 separate studies. Comprehensive Meta-Analysis software was used to determine differences based on time interval after burn, encompassing different phases of wound healing hemostasis, early and late inflammation, proliferation and early and late remodeling: post burn days (PBD)0-1, 2-4, 5-9, 10-14, 15-21, versus >21). The following categories we pre-defined for subgroup analyses: percentage of total body surface area (mild ≤5, moderate 5-25, versus severe >25), burn depth (superficial, partial-thickness, deep dermal, versus full-thickness), injury site (caudal, dorsal, lower body, both sides versus paw), burn agent (water, contact versus flame), species (human, mouse, rat versus pig), sex or age (young versus adult).

Common among-study variance across subgroups (pool within-group estimates of tau-squared) was assumed and subgroups were combined using fixed effects model. Effect was compared at different levels of the subgroups. Reported SMDs are based on the random effects model. P-values were based on the 95% confidence interval of the difference between subgroups. Bonferroni correction was applied, that is, the p-values were multiplied by the number of comparisons within each subgroup analysis.

### 2.9 Data visualization

Data was collected in Microsoft Excel (Microsoft (Microsoft Corp., Redmond, WA) and visualized using GraphPad version 8.0.2 (PRISM, Ja Jolla, USA).

## 3 Results

### 3.1 Study selection and study characteristics

**Figure 2:**
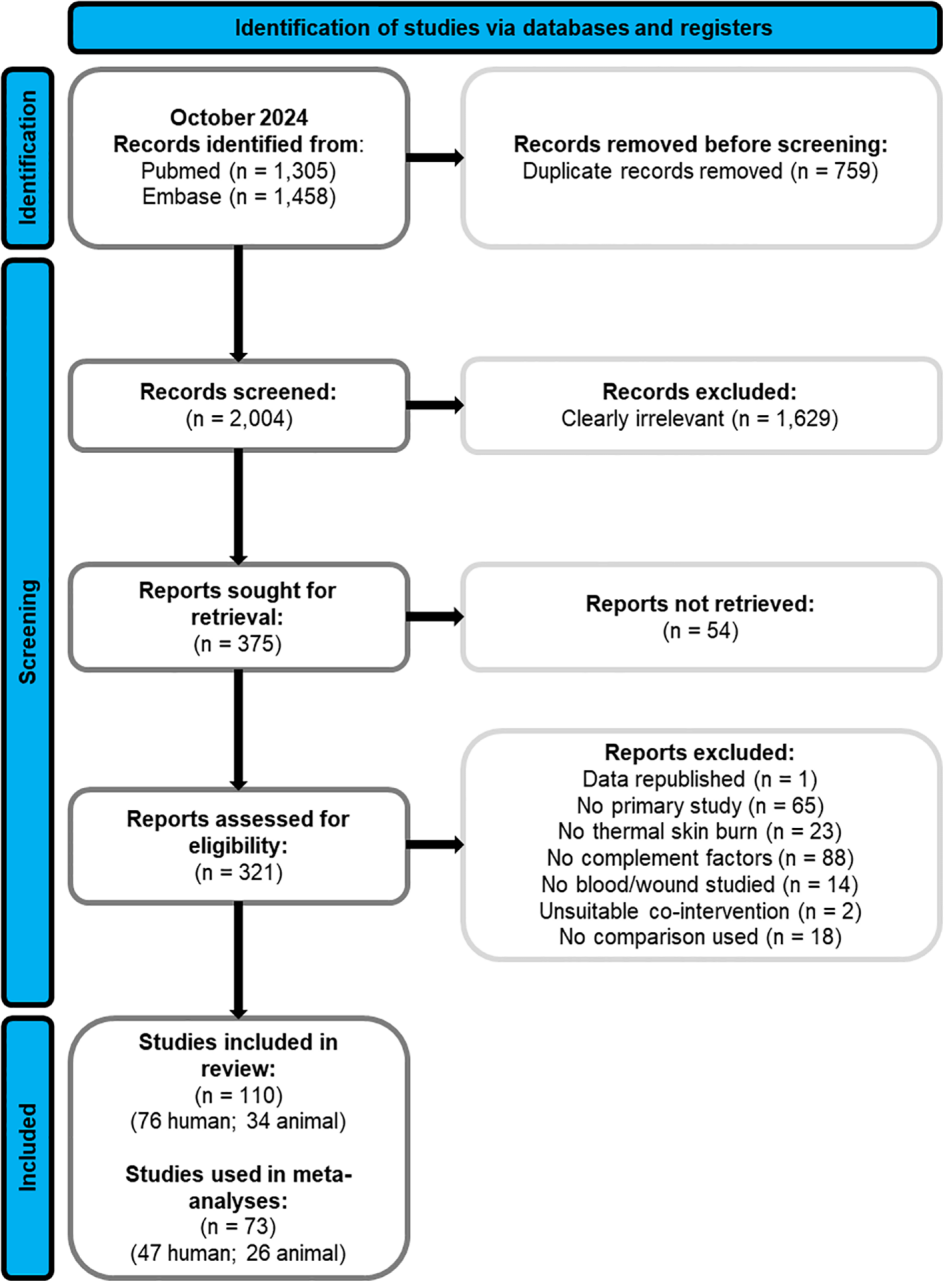
PRISMA flowchart illustrating the identification, screening, eligibility assessment and final inclusion of studies for the systematic review and meta-analyses (13).

The study protocol was pre-registered in PROSPERO (CRD420250510109). The systematic search yielded 2,004 records, of which 321 were included based on initial title and abstract screening (**Figure 2**). Full-text assessment was then performed on these studies; however, 54 articles could not be retrieved (**Table 1**). Ultimately, 110 studies met the predefined inclusion criteria and were incorporated into the systematic review, of which 73 contained sufficient and comparable data to be included in the meta-analyses (**Table 2**). The included studies comprised a range of different animal models (predominantly rats, followed by guinea pigs, mice, pigs, and dogs) and 76 human patient studies, with substantial heterogeneity in age, burn size, depth, and aetiology. While animal studies largely involved standardized, large full-thickness burns induced by water or flame, human studies showed wide variability in total body surface area, burn depth, and cause, with many characteristics incompletely reported (**Table 3**).

**Table 3:** Summary of study characteristics for animal and human studies.

Category		Animal (n)	Human (n)
Animal/human		35	76
Species	Dog	3 (1 Mongrel; 2 Beagle)	NA
	Guinea-pig	8 (8 Hartley)	
	Mouse	5 (2 C57BL/6; 1 CD1; 1 B6D2F1/J)	
	Pig	5 (2 Yorkshire; 2 not reported)	
	Rat	14 (5 Sprague-Dawley; 4 Long Evans; 3 Wistar; 1 SD, 1 Lewis)	
Age	Young	13	15
	Adult	5	32
	Mixed	0	21
	Not reported	17	8
TBSA	≤5	1	0
	5-20	6	0
	>20	26	22
	Mixed	0	52
	Not reported	2	2
Burn aetiology	Water	20	1
	Flame	8	5
	Contact	6	0
	Mixed	0	18
	Not reported	1	52
Burn depth	Superficial	0	0
	Partial-thickness	3	3
	Deep dermal	4	0
	Full-thickness	17	2
	Mixed	0	37
	Not reported	11	34
Burn location	Paw	1	0
	Dorsal	24	0
	Lower body	2	0
	Caudal	1	0
	Mixed	2	0
	Not reported	5	76

### 3.2 Study quality and risk of bias assessment

**Figure 3:**
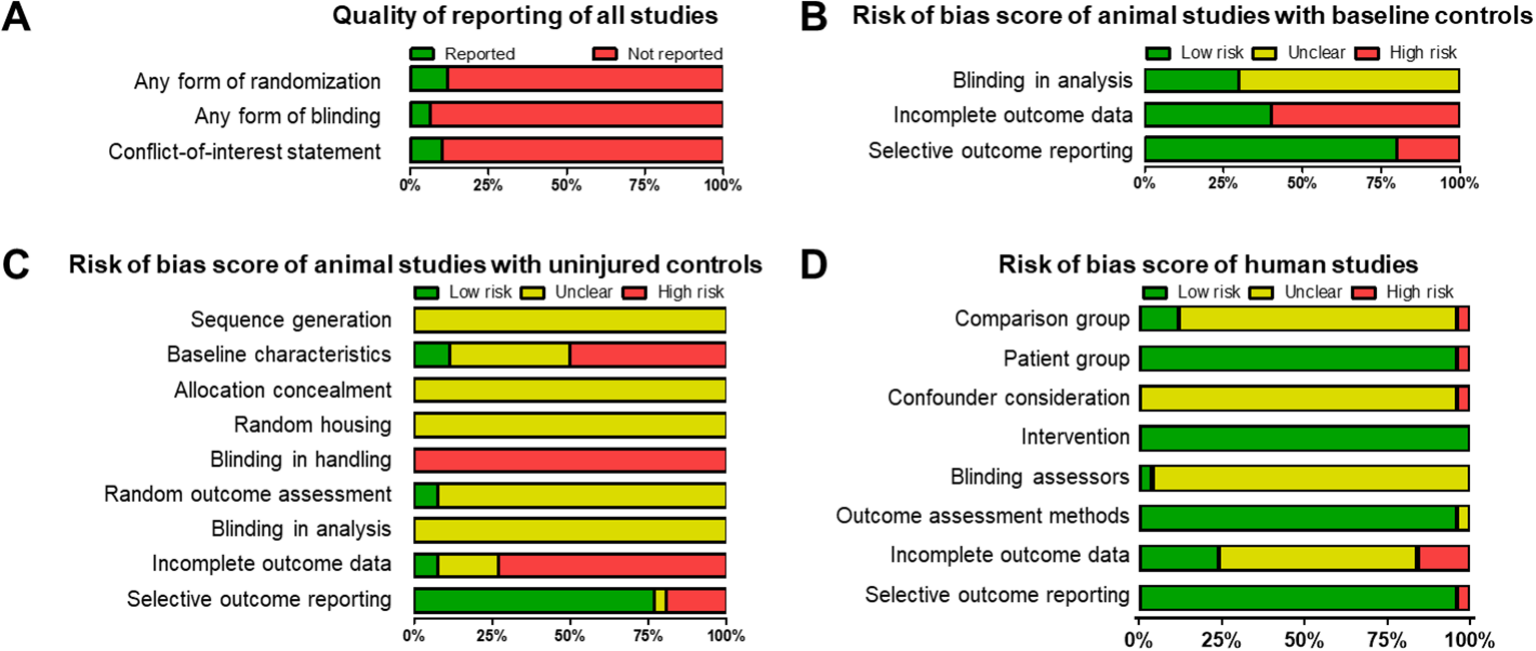
Quality of reporting and risk of bias assessment. **(A)** Quality of reporting of all included studies (n = 110). **(B)** Risk of bias assessment of all the included baseline-controlled animal studies (n = 10). **(C)** Complete risk of bias assessment of animal studies with an uninjured control group (n = 26). **(D)** Complete risk of bias assessment of human studies with an uninjured control group using ROBANS-II tool (n = 25) (16).

Substantial underreporting across studies hindered the evaluation of methodological quality. Most included studies failed to report fundamental design elements such as randomization procedures, blinding in analysis, or disclosure of conflicts of interest, resulting in a predominantly high or unclear risk of bias across assessed domains (**Figure 3A**).

Among the animal studies that used baseline (pre-burn) values as internal controls, approximately 30% explicitly reported some form of blinding during analysis, and about 40% sufficiently addressed incomplete outcome data. Notably, 80% did not demonstrate evidence of selective outcome reporting, suggesting that, despite other methodological limitations, most studies appeared to report all measured endpoints (**Figure 3B**).

In animal studies comparing burned animals with uninjured controls, none reported how randomization was applied during group allocation prior to burn induction (**Figure 3C**). Although random housing or blinding during handling is challenging or even impossible in such designs, the absence of reporting prevents meaningful assessment of selection bias. Blinding during outcome assessment or data analysis was also rarely described, and reporting on incomplete outcome data was often insufficient to determine whether attrition may have influenced results. Selective outcome reporting appeared common, with several studies failing to clarify why not all measured complement factors were reported.

Assessment of 25 randomly selected human studies using the ROBANS-II tool (**Figure 3D**) showed greater consistency in methodological quality but still revealed variability across domains. Most studies (n = 22; 88%) showed low risk of bias for patient group selection and outcome assessment methods (n = 23; 92%), indicating clearly defined cohorts and appropriate measurement techniques. However, confounder consideration (n = 19; 76% unclear risk), blinding of outcome assessors (n = 21; 84% unclear risk), and comparison group selection (n = 18; 72% high risk) frequently showed unclear or high risk owing to limited reporting or methodological constraints. Four studies (16%) demonstrated high risk related to incomplete outcome data, and 1 study (4%) showed high risk for selective outcome reporting.

### 3.3 Overall response of complement factors

**Figure 4:**
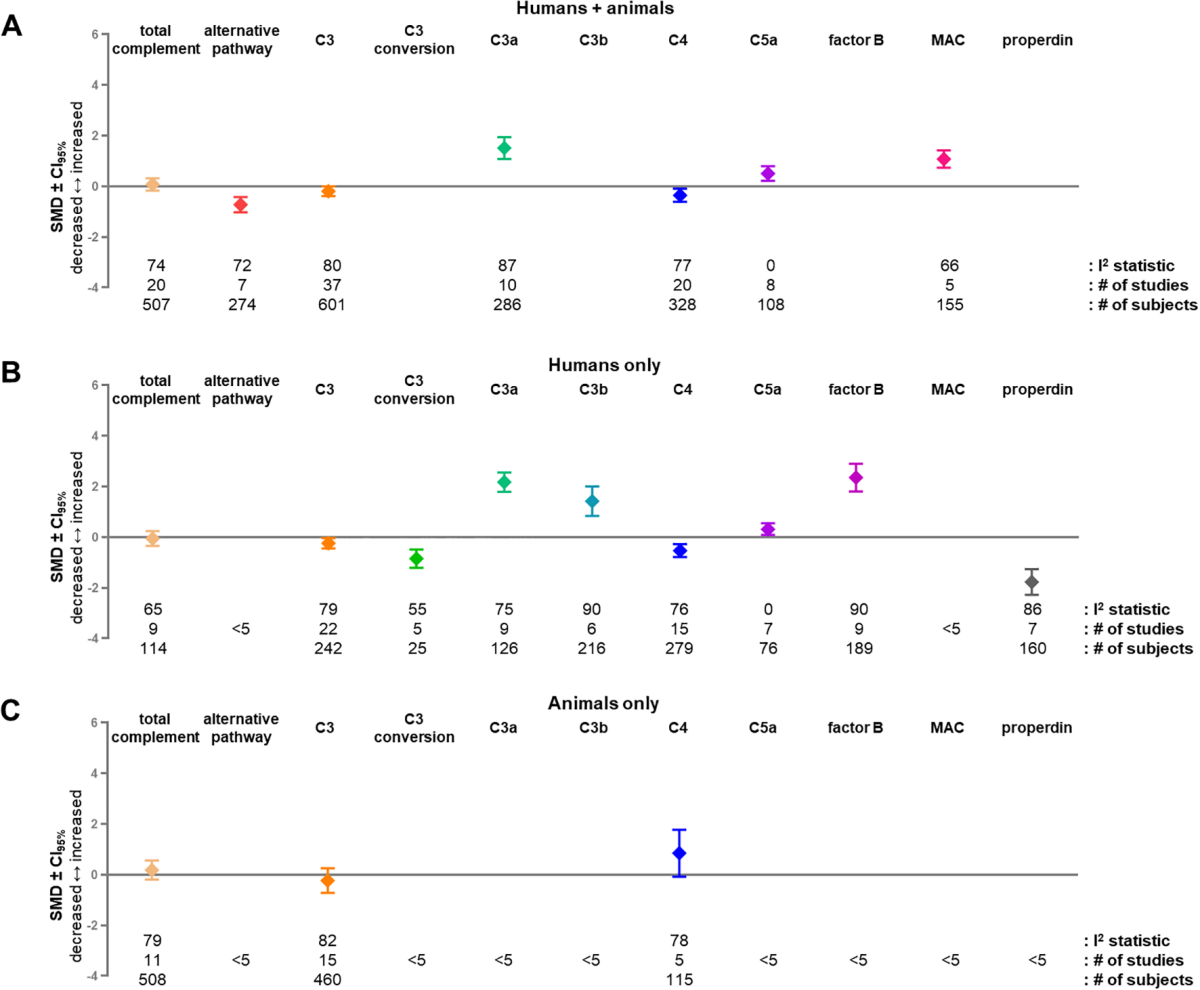
Overall level of complement factors after burn injury. Meta-analysis of the overall levels of complement factors in blood or burn wound tissue at any time after burn injury in humans and animals **(A)**, humans only **(B)** and animals only **(C)**. For C3 conversion, C3b, factor B and properdin only human studies were found. Only factors for which at least 5 separate studies were available are shown. Results are shown as SMD of levels of complement factors from burn-injured humans or animals compared to uninjured controls CI_95%_. There is a statistically significant difference with uninjured controls when the CI_95%_ does not cross the x-axis. The I^2^ statistic, number of studies and total number of subjects used in the burn-injured group for each meta-analysis are shown below the graphs. CI_95%_, 95% confidence interval; SMD, standardized mean difference.

Meta-analyses were performed for complement factors with data available from at least 5 different studies. Different sources (blood and wound), species (animal and human) and time-points were combined in the overall analysis. The analysis was performed on total complement (CH50/CH100), alternative pathway (AP50/AP100), C3, C3 conversion, C3a, C3b, C4, C5a, factor B, MAC, and properdin (**Figure 4A**). The overall analysis revealed a significant increase in factors C3a (SMD = 1.52; CI_95%_ = 1.09, 1.95; I^2^ = 87; n = 10 studies), C5a (SMD = 0.51; CI_95%_ = 0.22, 0.79; I^2^ = 0; n = 8 studies), and MAC (SMD = 1.08; CI_95%_ = 0.73, 1.43; I^2^ = 66; n = 5 studies) after burn injury, as compared to healthy controls. In contrast, alternative pathway (SMD = -0.72; CI_95%_ = -1.02, -0.42; I^2^ = 72; n = 7 studies), C4 (SMD = -0.35; CI_95%_ = -0.61, -0.10; I^2^ = 77; n = 20 studies), were decreased following burn injury. Total complement (SMD = 0.07; CI_95%_ = -0.17, 0.32; I^2^ = 74; n = 20 studies) and C3 (SMD = -0.19; CI_95%_ = -0.39, 0.00; I^2^ = 80; n = 37 studies) were not significantly altered after burn injury. Based on Duval and Tweedie’s trim-and-fill methodology, there was no indication that publication bias changed the direction of the observed effects. The adjusted SMD values were, however, significantly higher for C3b (SMD = 1.71; CI_95%_ = 1.54, 1.88) and factor B (SMD = 3.25; CI_95%_ = 3.11, 3.40) and significantly lower for properdin (SMD = -0.78; CI_95%_ = -0.95, -0.61).

### 3.4 Species-specific differences in complement activation after burn injury

Stratified analyses revealed both shared and divergent complement responses between human and animal studies (**Figure 4B**). In human studies, burn injury was associated with significantly increased levels of the activation products C3a (SMD = 2.18; CI_95%_ = 1.79, 2.56; I^2^ = 75; n = 9 studies), C3b (SMD = 1.42; CI_95%_ = 0.84, 2.01; I^2^ = 90; n = 6 studies), C5a (SMD = 0.32; CI_95%_ = 0.09, 0.54; I^2^ = 0; n = 7 studies) and factor B (SMD = 2.35; CI_95%_ = 1.81, 2.90; I^2^ = 90; n = 9 studies). In contrast, upstream components and functional readouts were reduced, including C3 (SMD = -0.24; CI_95%_ = -0.45, -0.04; I^2^ = 79; n = 22 studies), C3 conversion (SMD = -0.85; CI_95%_ = -1.21, -0.49; I^2^ = 55; n = 5 studies), C4 (SMD = -0.53; CI_95%_ = -0.79, -0.27; I^2^ = 76; n = 15 studies), and properdin (SMD = -1.77; CI_95%_ = -2.28, -1.26; I^2^ = 86; n = 7 studies). Total complement activity was not significantly altered in humans (SMD = -0.06; CI_95%_ = -0.35, 0.23; I^2^ = 65; n = 9 studies). Other components, including alternative pathway activity, MAC, and properdin, could not be analyzed separately in humans due to insufficient study numbers. In animal studies, effect estimates with no significant changes were observed for total complement activity (SMD = 0.19; CI_95%_ = -0.19, 0.56; I^2^ = 79; n = 11 studies), C3 (SMD = -0.23; CI_95%_ = -0.72, 0.25; I^2^ = 82; n = 15 studies) and C4 (SMD = 0.85; CI_95%_ = -0.08, 1.78; I^2^ = 78; n = 5 studies). However, direct comparison between species demonstrated a different effect direction and a significantly higher C4 response in animal studies compared with human studies (P = 0.0052).

Sensitivity analyses revealed significant species-dependent differences for several complement factors. Compared with animal studies, human studies showed markedly higher levels of C3a (animals: SMD = -1.34; CI_95%_ = -1.78, -0.89; n = 1 study versus humans: SMD = 2.18; CI_95%_ = 1.79, 2.56; n = 9 studies; P< 0.0001) and MAC (animals: SMD = 0.63; CI_95%_ = 0.36, 0.90; n = 2 studies versus humans: SMD = 1.98; CI_95%_ = 1.17, 2.78; n = 3 studies; P = 0.002). In contrast, C5a levels were significantly lower in humans than in animals (animals: SMD = 2.47; CI_95%_ = 1.14, 3.81; n = 1 study versus humans: SMD = 0.32; CI_95%_ = 0.09, 0.54; n = 7 studies; P = 0.002). No significant species differences were observed for alternative pathway activity or total complement.

### 3.5 Subgroup analyses at different time-intervals of complement factors after burn injury

**Figure 5:**
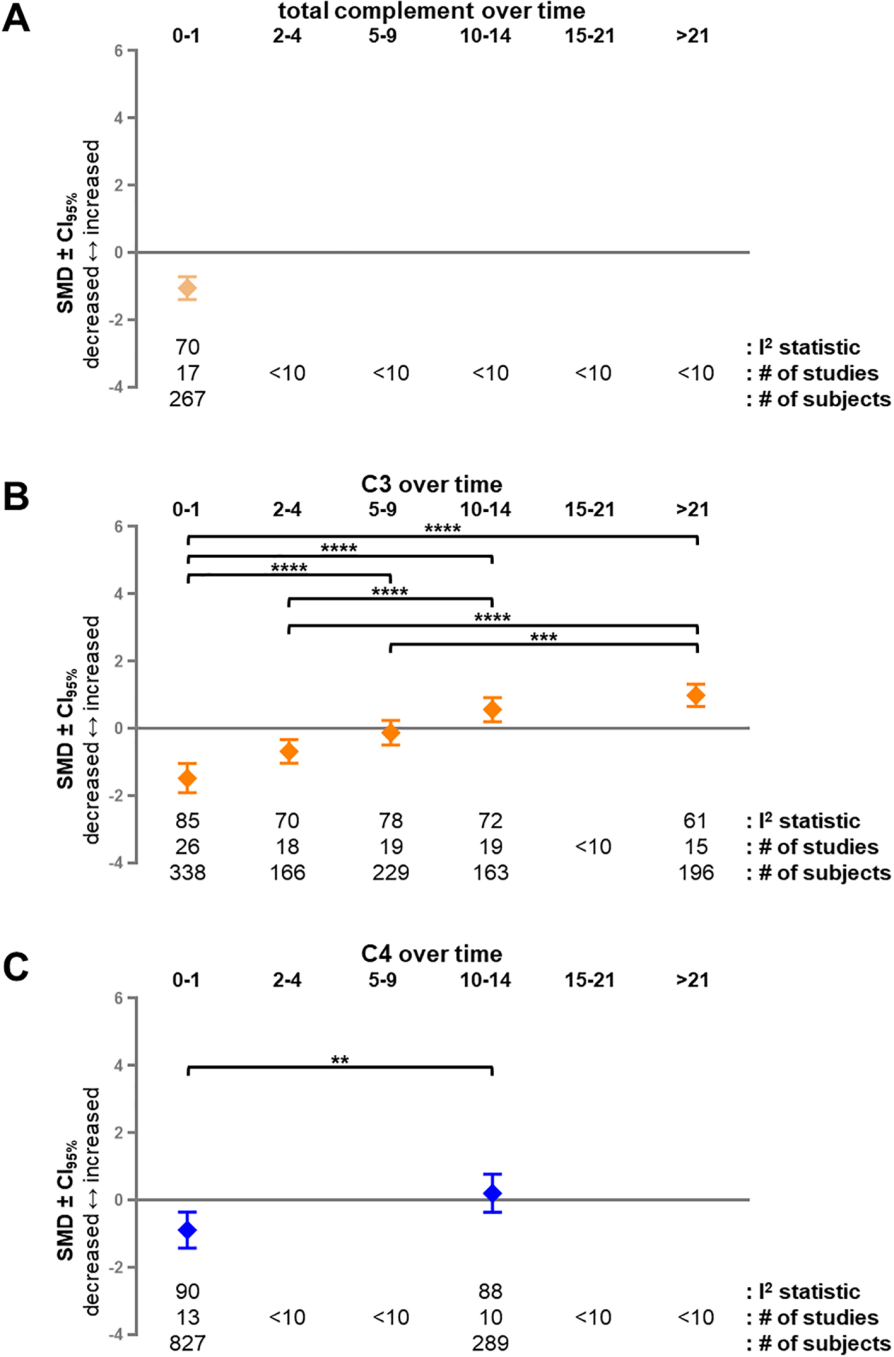
Longitudinal analysis of complement factors after burn injury. Subgroup analysis of different time intervals after burn injury for complement factors total complement **(A)**, C3 **(B)** and C4 **(C)** in blood or wound tissue. Only time intervals containing at least 10 separate studies were analyzed. Results are shown as SMD of levels of inflammatory mediators in burn-injured animals compared to uninjured animals CI_95%_. There is a statistically significant difference with uninjured animals when the CI_95%_ does not cross the x-axis. The I^2^ statistic, number of studies and total number of animals used in the burn-injured group for each meta-analysis are shown below the graphs. Significant Bonferroni-corrected P-values based on the CI_95%_ of the difference between time intervals are indicated by black asterisks: **P< 0.01; ***P< 0.001; ****P< 0.0001. CI_95%_, 95% confidence interval; SMD, standardized mean difference.

Next, the temporal dynamics of complement activation were assessed by performing subgroup analyses at different time intervals post-burn using the predefined threshold of 10 different studies per time interval. Due to the low number of studies that reported the same outcome measure, subgroup analysis could not be performed for percentage of total body surface area, burn depth, injury site, burn agent, species, sex or age. Temporal analyses could be performed for total complement hemolytic activity, C3, and C4 (**Figure 5**). Total complement was indifferent from control in the overall analysis (**Figure 4A**), but showed a significant early-phase reduction during the first 24 hours post-burn (SMD = -1.06; CI_95%_ = -1.40, -0.72; I^2^ = 70, n = 17 studies) (**Figure 5A**).

Temporal analysis of C3 levels revealed a distinct time-dependent pattern (**Figure 5B**). C3 was significantly reduced at post-burn day (PBD) 0-1 (SMD = -1.48; CI_95%_ = -1.92, -1.05; I^2^ = 91; n = 26 studies), remained decreased to at PBD2-4 (SMD = -0.69; CI_95%_ = -1.04, -0.34; I^2^ = 80; n = 18 studies), returned to healthy control levels at PBD5-9 (SMD = -0.13; CI_95%_ = -0.49, 0.24; I^2^ = 86; n = 19 studies), and was increased PBD10-14 (SMD = 0.56; CI_95%_ = 0.20, 0.92; I^2^ = 84; n = 19 studies) and beyond PBD21 (SMD = 0.99; CI_95%_ = 0.66, 1.32; I^2^ = 81; n = 15 studies). Bonferroni-corrected pairwise comparisons showed statistically significant differences between the different time intervals: levels at PBD0–1 were lower than at PBD5-9 (P< 0.0001), PBD10-14 (P<0.0001) and PBD>21 (P< 0.0001); levels at PBD2–4 were lower than at PBD10-14 (P< 0.0001) and PBD>21 (P< 0.0001); and levels at PBD5–9 were lower than at PBD>21 (P = 0.0006).

C4 levels were significantly reduced at PBD0-1 (SMD = -0.89; CI_95%_ = -1.43, -0.35; I^2^ = 90; n = 13 studies) (**Figure 5C**). At PBD10-14, C4 levels were indifferent from healthy control levels (SMD = 0.20; CI_95%_ = -0.37, 0.77; I^2^ = 88; n = 10 studies) and were significantly higher than at PBD0-1 (P = 0,0063). Collectively, these results indicate a time-dependent shift from early complement loss to later compensatory activation following burn injury.

### 3.6 Therapeutic interventions targeted at immune dysfunction after burn injury

An overview of all immune-related therapeutic interventions investigated in the included studies was generated (**Table 4**). This list of interventions displays the broad range of strategies explored to modulate complement activation and downstream immune disturbances following burn injury. Across the 110 included studies, 32 investigated therapeutic interventions related to complement modulation. These interventions were categorized into five groups: immunomodulatory approaches, complement pathway-directed treatments, mediators (cytokines, hormones and growth factors), nutritional interventions, and oxidative stress modulators. C3 was the most frequently measured complement outcome (28 studies, 88%), followed by total complement activity (11 studies, 34%). Studies were conducted predominantly in rats (18 studies, 56%), with additional mouse, guinea-pig, dog, and human studies.

**Table 4:** Therapeutic interventions studied within systematic review.

Category	Therapeutic	Species	Administration	Effect on studied factors	Reference
Immunomodulatory					
Antihistamines	Cimetidine-hcl, diphenhydramine-hcl	rat	i.p.	C3 (↑)	(51)
Macrophage supressors	Carrageenin	rat	i.v., i.p.	C5a (↓ 2h)	(42)
NSAIDs	Ibuprofen, indometacin, piroxicam	guinea-pig	i.m.	C3 (unclear)	(20)
	Ibuprofen	rat	injections	C3 (=)	(41)
Vaccinations	Vaccination C. Parvum	dog	i.v.	C3 (unclear)	(50)
Immunoglobulins	Prophylactic polyclonal IgG	human	i.v.	total complement (unclear)	(146)
Lymph node permeability suppressors	Anti-lymph-node-permeability-factor	rat	i.v.	alternative pathway (↑<48h) MAC (↑<96h) total complement (↑<4h)	(42)
Neutrophil depletors	Anti-neutrophil rabbit serum	rat	i.p.	C3 (unclear)	(51)
	Anti-neutrophil rat serum	rat	i.p.	C3 (=)	(44)
	Anti-polymorphonuclear serum	rat	i.v., i.p.	C3 (unclear)	(42)
	Anti-rat PMN antibody	rat	i.p.	C3 (unclear)	(35)
Complement pathway					
Complement inhibition	Antigen-antibody complexes, Heat-aggregated gamma-globulin	rat	i.v., i.p.	total complement (↓) C3 (↑) C4 (↑)	(42)
	Cobra venom factor	mouse	i.p.	C3 (=)	(27)
				C3 (=) C4 (=)	(26)
		rat	i.p.	C3 (=) total complement (=)	(44)
				C3 (=) total complement (=)	(35)
				C3 (↑ 48h) total complement (=)	(37)
				total complement (↑)	(51)
	C1 inhibitor	human	unclear	C3 (=)	(75)
	Heparin	rat	i.v.	C3 (=)	(52)
Plasma therapies	Fresh frozen plasma, plasma protein derivative	human	i.v.	C3 (unclear) total complement (=)	(54)
Soluble mediators					
Anabolic steroids	Oxandrolone	human	unclear	C3b (=) factor B (↑ 5–20 days) properdin (↑)	(64)
			enteral	C3 (↑) C3b (=)	(173)
Cytokines, antibodies	IgM, IL-18	mouse	i.p.	C3 (=)	(32)
Growth factors	IGF-1/BP-3	human	i.v.	C3 (↑)	(77)
	Growth hormone	human	i.m.	C3 (=) C4 (=)	(172)
Nutritional					
Feeding strategies	175 kcal/kg/day or 200 kcal/kg/day	guinea-pig	enteral	C3 (=) C3a (↑) C4 (↓ 3–6 days, 21–30 days)	(34)
	Bolus of feed or saline 3 hours PB	guinea-pig	enteral	C3 (↓ 23–40 days) MAC (↓)	(28)
	Bolus of feed or saline 12 hours PB	guinea-pig	enteral	C3 (↓23–34 days, 41–60 days) C3a (↑)	(28)
Lipids	N-3 Fatty acids	human	enteral	C3 (=) C4 (=)	(159)
Proteins and amino acids	15% kcal from casein/soy, hydrolyzed whey or whey	dog	enteral	total complement (unclear)	(49)
	Immediate or delayed feeding amino acid diet	guinea-pig	enteral	C3 (=)	(39)
	Immediate feeding whey protein	guinea-pig	enteral	C3 (↑)	(39)
	Milk whey protein supplementation	human	enteral	C3 (=)	(134)
	Glutamine	human	enteral	C3 (=)	(167)
Vitamins	Vitamin A	guinea-pig	enteral	C3 (unclear)	(155)
Oxidative stress					
Hydroxyl radical scavengers	DMSO, DMTU	rat	i.p.	C3 (=)	(44)
ROS scavengers	Catalase, SOD	rat	i.v.	C3 (unclear)	(51)
			i.v.	C3 (unclear)	(35)
	PEG-catalase	rat	i.v.	C3 (=)	(44)
Xanthine oxidase inhibitors	Allopurinol	rat	i.p.	C3 (unclear)	(21)
	Allopurinol, lodoxamine	rat	i.p., i.v.	C3 (unclear)	(35)

Thirty-two intervention studies categorized into five groups: immunomodulatory, complement pathway-directed mediators, nutritional, and oxidative stress modulators. Routes of administration: i.p., intraperitoneal; i.v., intravenous; i.m., intramuscular. Effect on studied factors is shown as significant change after burn as observed by the author.

## 4 Discussion

In this systematic review and meta-analysis, we combined human and animal studies to provide a comprehensive overview of post-burn complement activation. Across species, sample types, and study designs, we identified consistent increases in C3a, C3b, C5a, factor B, and MAC, while alternative pathway activity, C3 conversion products, C4, and properdin were reduced. Analyses over time revealed that C3 was reduced during the first 24 hours post injury, before becoming elevated at late time intervals. These findings indicate a dynamic complement response, with acute depletion followed by sustained activation of the complement system. Furthermore, the broad range of therapeutic strategies identified in our systemic review highlights the interest in targeting complement-mediated inflammation after burn injury.

The evidence base was dominated by human studies, providing strong clinical relevance, but substantial variability in injuries, populations, sampling time points, and analytical methods introduced high heterogeneity. Although this limited the feasibility of subgroup analyses, the aggregated data still revealed meaningful overall and temporal patterns that enhance understanding of burn-induced complement activation. Risk-of-bias assessments showed frequent gaps in reporting, particularly regarding experimental protocols, details about control samples, and the use of blinding. Publication bias was assessed using Duval and Tweedie’s trim and fill, and for none of the analyzed factors did publication bias alter the direction of the observed effect (**Supplementary File 2**). As trim-and-fill may be biased when applied to standardized mean differences due to small-study effects, results were interpreted cautiously and used only to assess whether publication bias altered the direction of effects (186). Human studies generally appeared to score better than animal studies, yet both categories suffered from incomplete methodological descriptions that restrict interpretability. As seen in previous reviews (187, 188), underreporting of essential details, such as intervention procedures, sample sizes, and measures of variance, also impeded meta-analysis. Failure to adhere to guidelines such as ARRIVE reduces data reusability, complicates assessment of study quality, and limits opportunities for advanced analyses. Strengthening reporting standards and ensuring access to raw data will improve research quality and reproducibility and ultimately support reduction, refinement, and replacement of animal models while accelerating translational progress in burn research (189–192).

Our meta-analysis revealed overall reduced levels of alternative pathway, C3 conversion, C4 and properdin, and reduced levels early after injury of total complement, C3 and C4, indicating acute complement consumption. Similar early depletion has been described in other trauma studies including polytrauma, where rapid activation leads to transient functional exhaustion before recovery (6, 193). This likely reflects abrupt recognition of damaged tissue through the classical and lectin pathways, combined with broad activation of the alternative pathway amplification loop (2). Functionally, this early depletion may compromise antibacterial defense by limiting opsonization and phagocytosis, contributing to the increased susceptibility to infection observed shortly after severe burns. As complement proteins are replenished during the acute-phase response, the balance shifts toward sustained and excessive complement activation, as shown by substantial increases in C3a, C3b, C5a, factor B, and MAC levels. This late rise of complement aligns with prolonged systemic inflammation, tissue injury and impaired wound healing often seen after major burn trauma (1, 194, 195). The later phase of complement activation is closely aligned with prolonged systemic inflammation, endothelial dysfunction, and tissue injury. High levels of anaphylatoxins such as C5a can promote leukocyte recruitment while simultaneously impairing neutrophil function through receptor desensitization, fostering a state of inflammatory yet ineffective host defense (156). In parallel, increased MAC formation may contribute to secondary tissue damage, burn wound progression, and distant organ injury (193). Together, these findings support a model in which early complement consumption may predispose to immune vulnerability, followed by prolonged complement-driven inflammation, although direct causal links with clinical outcomes remain primarily supported by experimental models.

Translating findings from animal models to the human burn setting remains a major challenge, particularly when accounting for factors such as age, burn type, severity, and comorbidities that influence clinical trajectories (196). Although animal studies have historically informed our understanding of complement activation after burns, the current literature is dominated by human data. Still, extrapolation from animals to patients is complicated by fundamental physiological differences, including immune signaling, wound contraction, and scar formation (197–199). Combining human and animal data is further complicated by differences in study design: animal experiments use standardized burn models with controlled conditions, whereas human studies display substantial heterogeneity in injury characteristics and clinical management. Although differences between individual animal species may influence complement responses after burn injury, the number of available studies per species was insufficient to permit statistically robust subgroup analyses. Despite these limitations, animal studies, though few in number, showed similar overall trends in total complement, C3, and C4 compared with human studies. Sensitivity analyses further indicated conserved patterns of early complement consumption followed by subsequent activation across species, while revealing species-specific differences in effect magnitude for certain components, including C3a, C5a, and MAC. Importantly, the consistent observation of early complement depletion followed by later overactivation across both species suggests that key aspects of complement response to burn injury are conserved between humans and rodents.

The biphasic pattern of complement dysregulation observed after burn injury indicates that complement modulation may represent a promising but time-dependent therapeutic strategy. Early complement consumption may contribute to transient immune vulnerability, whereas the later and sustained increase in C3a, C5a, factor B, and MAC suggests ongoing complement-driven inflammation. In particular, elevated C5a is a potent driver of neutrophil recruitment and activation, and persistent exposure can lead to both hyper-inflammatory responses and neutrophil dysfunction through receptor desensitization, resulting in ineffective host defense despite ongoing inflammation (200). Therapeutic approaches could therefore focus on selectively attenuating excessive complement activation, such as inhibition of C5 or C5a–C5aR signaling, modulation of alternative pathway amplification, or restoration of complement regulation, during the later post-burn phase while preserving antimicrobial functions. However, clinical studies specifically evaluating complement-targeted interventions in burn patients remain limited, and although current evidence provides only indirect insights into efficacy, dosing, and timing, the consistent patterns identified support further investigation while carefully balancing anti-inflammatory effects with preservation of antimicrobial defense. Future research should prioritize standardized longitudinal sampling and biomarker-guided patient stratification to define optimal therapeutic windows and evaluate whether complement modulation can reduce inflammation, limit secondary tissue damage, and improve wound healing and clinical outcomes after severe burn injury.

This systematic review and meta-analysis provide, to our knowledge, one of the most comprehensive synthesis to date of complement activation after burn injury, integrating data from both human and animal studies. Key strengths include the preregistered protocol, broad and systematic search strategy, and the use of meta-analysis. Several limitations should be considered when interpreting these findings. First, substantial heterogeneity was present across studies with respect to burn severity, sampling strategies, and reporting quality, which limited the feasibility of detailed subgroup analyses beyond time after injury. However, the between study heterogeneity was partly taken into account by using the random effects model. Notably, heterogeneity for C5a was low (I² = 0%), indicating no detectable between-study variability beyond sampling error, although this should be interpreted with caution given the limited number of studies. Second, underreporting of essential methodological details, including randomization, blinding, control selection, and measures of variance, was common in both animal and human studies and contributed to a generally high or unclear risk of bias. Incomplete reporting and missing data led to the exclusion of a considerable number of otherwise relevant studies from meta-analysis. This may have hampered the certainty of the evidence as the precision of the meta analytic analyses may have been decreased. Finally, while human studies dominated the evidence base and enhance clinical relevance, many lacked standardized outcome definitions and adjustment for confounding factors, whereas animal studies often relied on simplified burn models that may not fully capture the complexity of human burn physiology. Together, these limitations highlight the need for improved reporting standards, and open access to raw data to enhance reproducibility, reusability, and translational value of future burn research.

In conclusion, this systematic review and meta-analysis show that burn injury triggers a dynamic complement response. An early phase of complement consumption, reflected by reduced total complement activity, C3 and C4, is followed by sustained activation with increased C3a, C3b, C5a, factor B and MAC. This dynamic shift likely reflects initial depletion due to overwhelming activation, followed by prolonged complement-driven inflammation. These patterns indicate that the complement response to burn injury may contribute to systemic inflammation, impaired wound healing, and poorer outcomes. Despite the predominantly descriptive nature of the evidence, the complement system remains as a promising therapeutic target. Future work should prioritize standardized longitudinal sampling, comprehensive profiling of complement components, and time-specific evaluation of complement-modulating interventions to guide effective clinical translation.

## Data Availability

The original contributions presented in the study are included in the article/**Supplementary Material**. Further inquiries can be directed to the corresponding author.
