# Supplementary material for: Complement response to burn injury: systematic review and meta-analysis of patient and animal studies
Source: Front Immunol. 2026 Feb 25;17:1793945. doi: 10.3389/fimmu.2026.1793945 (PMC12975936; doi:10.3389/fimmu.2026.1793945)
Supplement: Supplementary file 1 [file Supplementaryfile1.docx]

**Search strategy Pubmed (Medline)**

**Search Component 1: (**Burns[MeSH] OR burns[tiab] OR burn[tiab] OR burnt[tiab] OR burned[tiab] OR scald[tiab] OR scalds[tiab] OR thermal injur*[tiab] OR thermal wound*[tiab] OR heat injur*[tiab] OR heat wound*[tiab])

**Search Component 2: (**Complement System Proteins[MeSH] OR complement*[tiab] OR membrane attack complex[tiab] OR MAC[tiab] OR convertase*[tiab] OR anaphylatoxin*[tiab] OR C1[tiab] OR C1r[tiab] OR C1s[tiab] OR C1q[tiab] OR C1inh*[tiab] OR C1-inh*[tiab] OR C2[tiab] OR C2a[tiab] OR C2b[tiab] OR C3[tiab] OR C3a[tiab] OR C3b[tiab] OR C4[tiab] OR C4a[tiab] OR C4b[tiab] OR C5[tiab] OR C5a[tiab] OR C5b[tiab] OR C6[tiab] OR C7[tiab] OR C8[tiab] OR C9[tiab] OR factor B[tiab] OR factor D[tiab] OR factor I[tiab] OR factor H[tiab] OR properdin*[tiab] OR Decay-accelerating factor[tiab] OR protectin[tiab] OR CD59[tiab] OR membrane cofactor protein[tiab] OR CD46[tiab] OR MCP[tiab])

**Search strategy Embase**

**(**burn/exp OR burns:ab,ti,kw OR burn:ab,ti,kw OR burnt:ab,ti,kw OR burned:ab,ti,kw OR scald:ab,ti,kw OR scalds:ab,ti,kw OR ‘thermal-injur’*:ab,ti,kw OR ‘thermal-wound’*:ab,ti,kw OR ‘heat-injur’*:ab,ti,kw OR ‘heat-wound’*:ab,ti,kw)

AND

(complement/exp OR complement:ab,ti,kw OR ‘membrane attack complex’:ab,ti,kw OR MAC:ab,ti,kw OR convertase*:ab,ti,kw OR anaphylatoxin*:ab,ti,kw OR C1:ab,ti,kw OR C1r:ab,ti,kw OR C1s:ab,ti,kw OR C1q:ab,ti,kw OR C1inh*:ab,ti,kw OR ‘C1-inh’*:ab,ti,kw OR C2:ab,ti,kw OR C2a:ab,ti,kw OR C2b:ab,ti,kw OR C3:ab,ti,kw OR C3a:ab,ti,kw OR C3b:ab,ti,kw OR C4:ab,ti,kw OR C4a:ab,ti,kw OR C4b:ab,ti,kw OR C5:ab,ti,kw OR C5a:ab,ti,kw OR C5b:ab,ti,kw OR C6:ab,ti,kw OR C7:ab,ti,kw OR C8:ab,ti,kw OR C9:ab,ti,kw OR ‘factor B’:ab,ti,kw OR ‘factor D’:ab,ti,kw OR ‘factor I’:ab,ti,kw OR ‘factor H’:ab,ti,kw OR properdin*:ab,ti,kw OR ‘Decay-accelerating factor’:ab,ti,kw OR protectin:ab,ti,kw OR CD59:ab,ti,kw OR ‘membrane cofactor protein’:ab,ti,kw OR CD46:ab,ti,kw OR MCP:ab,ti,kw)
